# Expedite SERS Fingerprinting of Portuguese White Wines Using Plasmonic Silver Nanostars

**DOI:** 10.3389/fchem.2019.00368

**Published:** 2019-05-24

**Authors:** Miguel Peixoto de Almeida, Nicolae Leopold, Ricardo Franco, Eulália Pereira

**Affiliations:** ^1^LAQV, REQUIMTE, Departamento de Química e Bioquímica, Faculdade de Ciências da Universidade do Porto, Porto, Portugal; ^2^Faculty of Physics, Babeş-Bolyai University, Cluj-Napoca, Romania; ^3^UCIBIO, REQUIMTE, Departamento de Química, Faculdade de Ciências e Tecnologia, Universidade NOVA de Lisboa, Caparica, Portugal

**Keywords:** silver nanostars, surface-enhanced Raman spectroscopy (SERS), white wine, authenticity test, fingerprinting, principal component analysis (PCA)

## Abstract

Surface-enhanced Raman Spectrosocopy (SERS) is a highly sensitive form of Raman spectroscopy, with strong selectivity for Raman-active molecules adsorbed to plasmonic nanostructured surfaces. Extremely intense Raman signals derive from “hotspots”, generally created by the aggregation of a silver nanospheres colloid. An alternative and cleaner approach is the use of anisotropic silver nanoparticles, with intrinsic “hotspots”, allowing a more controlled enhancement effect as it is not dependent on disordered nanoparticle aggregation. Here, a simple SERS-based test is proposed for Portuguese white wines fingerprinting. The test is done by mixing microliter volumes of a silver nanostars colloid and the white wine sample. SERS spectra obtained directly from these mixtures, with no further treatments, are analyzed by principal component analysis (PCA), using a dedicated software. Depending on the duration of the incubation period, different discrimination can be obtained for the fingerprinting. A “mix-and-read” approach, with practically no incubation, allows for a simple discrimination between the three white wines tested. An overnight incubation allows for full discrimination between varieties of wine (*Verde* or *Maduro*), as well as between wines from different *Maduro* wine regions. This use of SERS in a straightforward, fast and inexpensive test for wine fingerprinting, avoiding the need for prior sample treatment, paves the way for the development of a simple and inexpensive authenticity assay for wines from specific appellations.

## Introduction

The gold standards for wine characterization are chromatography methods, especially liquid chromatography. Methods such as liquid chromatography coupled to mass spectroscopy (LC–MS) and high-performance liquid chromatography (HPLC) are typically used, and efficient, but preliminary fractionation and purification steps are often needed before any analytical procedure (Carpentieri et al., 2007). Wine phenolic fraction is typically analyzed by these chromatographic methods.

Several techniques were already described for wine discrimination based on the statistical analysis of their outputs (typically spectra). Red wines were analyzed by fluorescence spectroscopy and data analysis by parallel factor analysis (PARAFAC) allowed to distinguish wines from different world regions (e.g., Australia, Chile, Spain, USA) (Airado-Rodríguez et al., 2009). A group of four techniques were compared for the same batch of red wines (from only two different appellations): near infrared spectroscopy (NIR), ultraviolet-visible spectroscopy (UV-Vis), a headspace-mass “artificial nose” and a voltammetric “artificial tongue” (Casale et al., 2010). The best separation, determined by principal component analysis (PCA) and linear discriminant analysis (LDA), was achieved by the spectroscopic techniques, which is excellent, given their speed and ease of use. White wines were also discriminated by MALDI–MS (Rešetar et al., 2016), and by a combination of NIR, MIR and Raman spectroscopies (Teixeira dos Santos et al., 2017).

Phenolic composition is one of the most important quality parameters of wines. This composition is directly related to wine organoleptic characteristics such as color, astringency, and bitterness (Paixão et al., 2007). The resonance condition of phenolic compounds, such as hydroxycinnamic acids, has been analyzed selectively by Raman spectroscopy, using lasers at different wavelengths (Martin et al., 2015). Also by Raman spectroscopy, a complete chemometric analysis was implemented to discriminate different wines from the protected designation of origin of Piemonte (Northwest Italy) in accordance with grape varieties, production area and aging time (Mandrile et al., 2016), or to discriminate between wine variety (Feteasca Regala and Sauvignon Blanc) and geographic origin of Romanian wines. These works are relevant examples of the development of analytical methods for traceability to authenticate the geographical origin of foods, a mandatory requirement in the European Union (Peres et al., 2007; Regulation, 2012).

Surface-enhanced Raman spectroscopy (SERS) is an easy, simple, and rapid spectroscopic detection technique, with the possibility of portable use. Unlike Raman, SERS is selective to molecules adsorbed to metal nanostructures, leading to a drastic increase in the intensity of Raman signals originating from those specifically adsorbed molecules. This leads to molecular fingerprint specificity, high sensitivity, and narrow spectral bandwidth, making SERS highly promising for identifying disease markers from complex mixtures of clinical samples (Sinha et al., 2016), antibiotic-resistant bacteria (Galvan and Yu, 2018; Wang et al., 2018), viruses (Paul et al., 2015), or pathogenic fungi (Witkowska et al., 2018), to name a few examples in the medical area. In food analysis, SERS one-pot analysis is especially interesting in areas where inexpensive assays are necessary. SERS fingerprinting followed by PCA analysis has been successfully applied to discriminate tea varieties (Buyukgoz et al., 2016), for example, but never, to our knowledge, to discriminate wines from different regions.

There are two main varieties of Portuguese wines, namely, *Verde* and *Maduro*. *Verde* wine is typical from Northwest wine region of Portugal (Fraga et al., 2014). Across the remaining territory, *Maduro* wine is essentially produced, original from areas like *Douro* (Northeast) and *Alentejo* (South) wine regions. The main difference between *Verde* and *Maduro* wines is essentially the harvesting time, which is at an earlier stage of maturation for *Verde* wine. Hence the names in Portuguese language, “Verde” meaning “green” and “Maduro” meaning “ripe” (Fraga et al., 2014). This incomplete maturation stage influences the profile of compounds responsible for the organoleptic properties of wine. Between the several *Maduro* wine regions the main differences are due to the climate conditions. For example, *Douro* region has a lower average temperature and higher precipitation levels when compared to the *Alentejo* region (Fraga et al., 2014, 2015; Cunha and Richter, 2016). These factors are expected to influence the chemical composition of wines.

Our main goal was to fingerprint white wines from three different wine regions in Portugal using a simple SERS-based method, that could be ultimately used in a point-of-need assay to authenticate the origin of the particular wine tested. Silver nanostars were used as signal enhancers. Silver nanostars (AgNSs) are probably the most interesting anisotropic nanoparticles for SERS applications. In fact, AgNS superior plasmonic properties and remarkable capacity to form “hotspots”, make them ideal substrates for adsorption of Raman-active species. The multiplicity or number of arms, arm's length and resulting global size enables interaction with light across all the visible spectrum, although with higher intensity at the ~400 nm region (Garcia-Leis et al., 2013). Furthermore, AgNS fulfill all the requirements regarding the typical morphology that favors “hotspots”, namely, rough surface, sharp tips, intra- and interparticle nanogaps (Liu et al., 2016). Some of us have already proved the high SERS performance of these particular AgNS, for trace analyte detection on paper substrates (Oliveira et al., 2017).

To avoid laborious and/or costly sample analysis, including pre-treatment processes, spectra were analyzed by PCA, a projection method that provides the best fit of data distribution (Biancolillo and Marini, 2018). This fingerprinting technique is much more expedite than the more common analysis of specific wine components, implying separation and pre-concentration procedures, before applying the sample on SERS substrates (Chen et al., 2016; Li et al., 2016).

**Figure 1:**
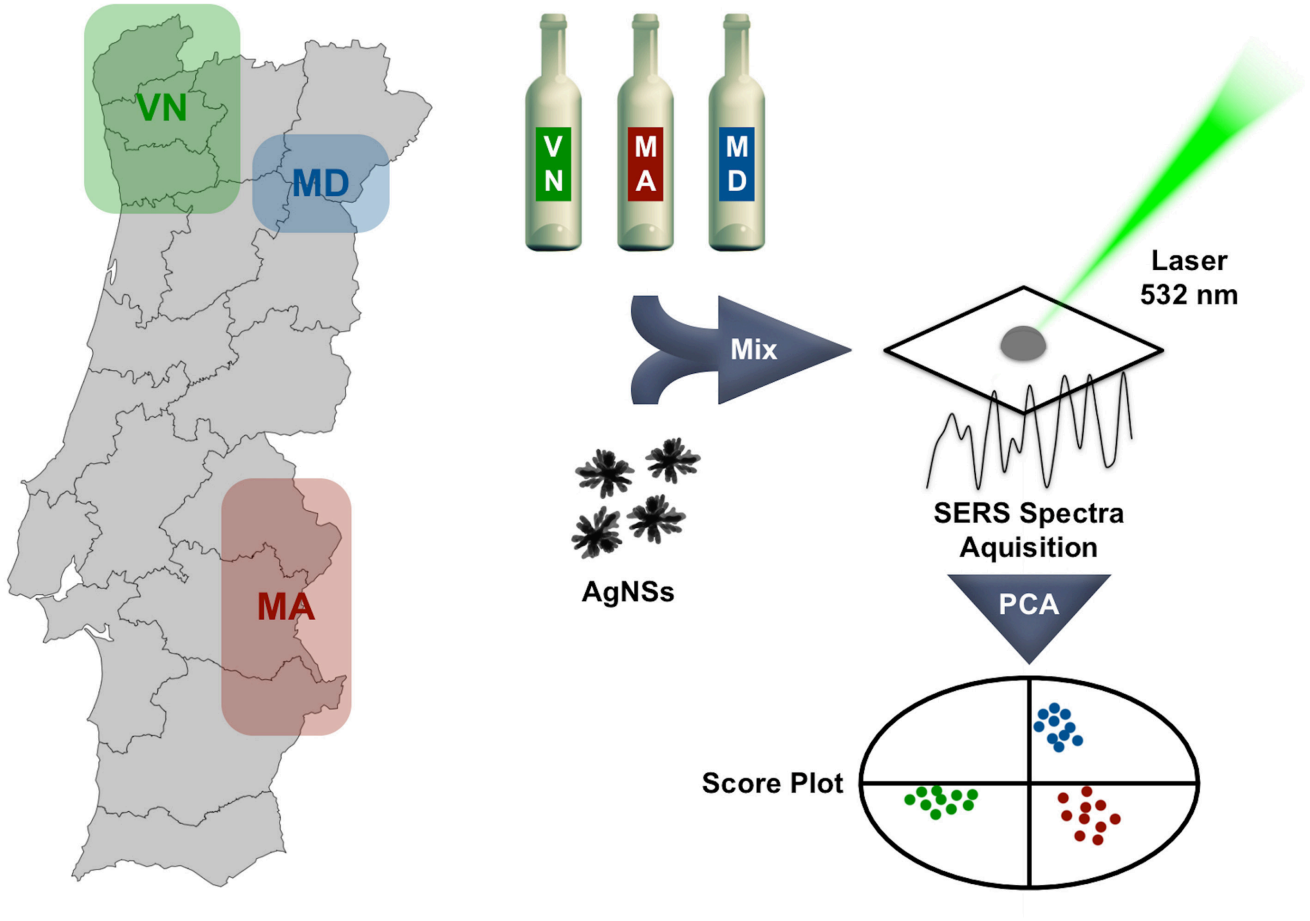
White wine samples from three different wine regions in Portugal are simply mixed with a silver nanostars colloid and incubated. A 532 nm laser is used to obtain the respective SERS spectra, that after principal component analysis (PCA) produces a score plot allowing sample discrimination. The duration of the incubation period is determinant for the obtained results.

Figure 1 depicts our approach to an expedite method for white wine fingerprinting.

## Materials and Methods

All the reagents were used as purchased, with no further steps of purification. For nanoparticle synthesis, a KD Scientific KDS 200 syringe pump, holding a BD 60 mL plastic syringe was used. Centrifugations were in a Sigma 30K centrifuge, with a 19776H rotor. Ultraviolet-visible spectroscopy (UV-Vis) was performed using a Varian Cary 50 Bio spectrophotometer. Nanoparticle tracking analysis (NTA) was using a Malvern Nanosight NS300 equipped with a 642 nm (red) laser module, by acquisition of 5 videos of 1 min each in static mode. Transmission electron microscopy (TEM) was performed using a Hitachi H8100 transmission electron microscope.

### Wine Samples

Portuguese white wine samples were chosen from two different varieties, *Verde* and *Maduro*. From the latter variety, wines from two different regions were tested. One commercial bottle, representative of each wine type was used. The wines were all bottled in 2014. The storage and aging processes of the specific wines are undisclosed, but it is known that *Verde* wine is stored in stainless steel containers, whereas *Maduro* wine is stored in wood barrels. Experimental protocols were applied to two independent samples of each bottle. Each sample was read in 5 different points, in a total of 10 spectra for each wine. The following abbreviations are used to designate the samples under study: *Verde* wine from the *Northwest* region (VN), *Maduro* wine from the *Douro* region (MD), and *Maduro* wine from the *Alentejo* region (MA). Wines were used as direct samples, with no further treatment.

### Preparation of Silver Nanostars

Star-shaped silver nanoparticles were synthesized based in the method from Garcia-Leis *et al* (Garcia-Leis et al., 2013). The following reagents were used: silver nitrate 99.9999% (Aldrich), hydroxylamine solution 50% wt. in water 99.999% (Aldrich), sodium hydroxide 98% (Fisher) and trisodium citrate dihydrate 99.0% (Merck). All glassware was previously treated with *aqua regia* and rinsed abundantly with deionized water, followed by ultrapure water (18.2 MΩ·cm at 25°C). All solutions were prepared with ultrapure water.

For the synthesis reaction, 2.5 mL of a 50 mM sodium hydroxide solution (125 μM) and 2.5 mL of a 60 mM hydroxylamine solution (150 μM) were mixed in beaker and, immediately after, 45 mL of a 1 mM silver nitrate solution (45 μM) was added dropwise from a syringe, using a syringe pump at a 45 mL/min injection rate. After 90 s, 500 μl of a 1.5% wt. trisodium citrate (dihydrate) solution was added to the mixture. The reaction vessel was kept protected from light for a 3-h reaction period.

After this 3-h period, the content of the five beakers was mixed and the resultant batch (250 mL) of AgNSs suspension was centrifuged for 12 min at a relative centrifugal force (RCF) of 1,600 g. The pellet was resuspended in ultrapure water (up to 10% of the initial volume) and stored in a glass vial.

### Raman and SERS Experiments

Raman and SERS spectra were measured with a Renishaw InVia Raman microscope, coupled with 442 nm 80 mW (He-Cd), 532 nm 200 mW (diode), 633 nm 17 mW (He-Ne), 785/830 nm 300 mW (diode) lasers. Gratings of 1,200 l/mm and 1,800 l/mm were used, the first for 785 nm measurements and the second for 442 nm, 532 nm and 633 nm. The system is equipped with a CCD camera detector, with a 1,040 × 256 resolution. The objective used had a 50x magnification.

Incubation protocol: 1 ml of wine and 100 μl of 0.1 nM AgNS colloid were mixed, followed by overnight incubation. Immediately before Raman/SERS measurements, samples containing nanoparticles were centrifuged for 5 min at RCF 1,600 g, and 99% of the supernatant volume was removed. The pellet rich in AgNS with adsorbed wine components, was dispersed in the remaining supernatant by “up and down” pipetting. From this final volume, 10 μl aliquots for analysis were taken. As a control experiment, Raman and SERS measurements were made of the supernatant fraction, with no detectable signals for any of the measurements.

“Mix-and-read” protocol: 10 μl of wine and 10 μl of 0.1 nM AgNS colloid were mixed and read immediately. No concentration steps were needed for this protocol, since the final volume was already 20 μl. All measurements were also performed in 10 μl aliquots.

Aliquots were placed in a microscope glass slide, wrapped in one layer of commercial-grade aluminum foil. When justifiable, spectra of the external elements to the sample, such as the aluminum foil, were acquired. The aluminum foil is in the origin of some peaks found in spectra collected with 633 nm and 785 nm lasers (Supplementary Figure 1), and one of the reasons why data obtained with these lasers was further excluded (see below).

Signal acquisition parameters such as laser power and acquisition time were different for the two protocols, with batches of spectra analyzed together acquired under identical experimental conditions. Number of acquisitions was always two per sample and acquisition times were between 4 and 10 s.

### Selection of Laser Line for SERS

In order to choose the appropriate laser line for maximum spectral intensity and resolution, SERS spectra were obtained for the three white wines mixed with AgNS colloid, following the overnight incubation protocol, using four different laser lines: 442, 532, 633, and 785 nm (Supplementary Figure 2). Spectra collected with the blue laser line (442 nm), are poorly structured, not allowing an easy fingerprinting of the wine samples. When the 532 nm laser line was used to obtain SERS spectra of this set of samples, it was possible to observe well-defined vibrations that may be helpful to the fingerprinting. Spectra acquired with the near infrared laser (785 nm line) presents sharp peaks, for the MD sample (as also observed for the 633 nm and 532 nm laser lines), but presents broad peaks leading to less defined spectra for the other two samples (MA and VN). A practical difficulty that contributed for the decision to exclude analysis based on the 633 or 785 nm laser lines, was the interference of the sample substrate observed for spectra obtained with these two laser lines (see Supplementary Figure 1). In conclusion, spectra obtained with 532 nm laser line are the most structured and, therefore, richer in information, being this one the selected laser line for these SERS studies. Also, using the 532 nm laser line in combination with the 1,800 lines/mm grating, enables the possibility of collecting data, in single measurement mode, in a larger range (~1,750 units of Raman shift) than 633 or 785 nm laser lines. This lower resolution for the 532 nm laser line does not seem to compromise data quality.

### Principal Component Analysis

Principal component analysis (PCA) was performed using The Unscrambler® X 10.4.1 (CAMO) software. For PCA analysis, ten spectra were collected per wine sample. Five spectra were collected in five different spots in both duplicates. Before application of PCA, spectra were submitted to data pre-treatment by baseline linear correction (BLC). Spectra were considered in the full range 100-1,800 cm^−1^.

## Results and Discussion

**Figure 2:**
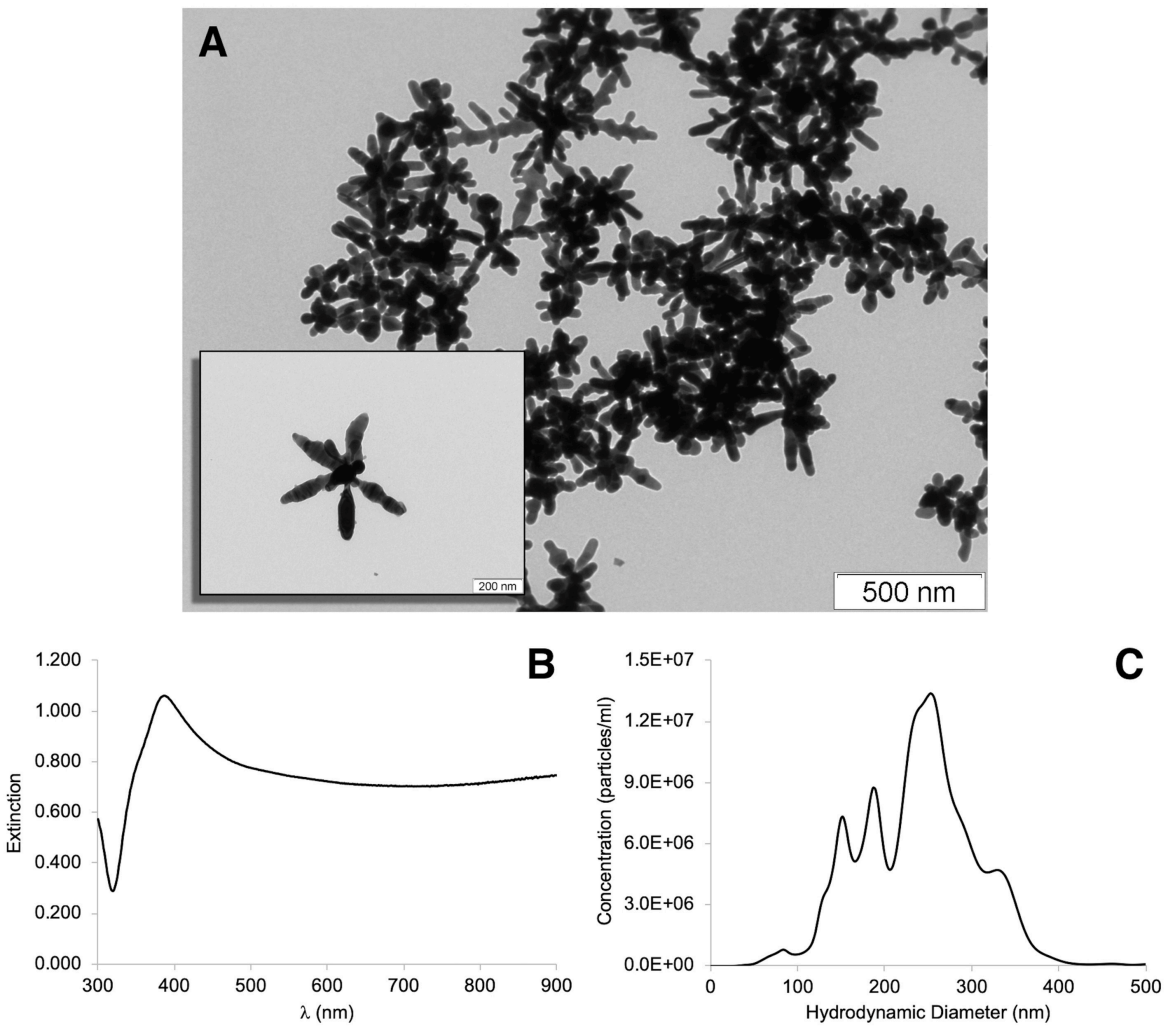
Silver nanostars (AgNS) colloid characterization by **(A)** Transmission Electron Microscopy (TEM) micrographs; **(B)** UV-visible spectrum, and **(C)** hydrodynamic diameter distribution, obtained by Nanoparticle Tracking Analysis (NTA).

Silver nanostars characterization was made using Transmission Electron Microscopy (TEM), ultraviolet-visible spectroscopy (UV-Vis) and nanoparticle tracking analysis (NTA) (Figure 2). TEM micrographs depicted in Figure 2A show star-shaped nanoparticles, with variable tip to tip size consequence of the number, relative position and shape of the arms. Each one of the nanoparticles is noticeably unique. The UV-vis spectrum depicted in Figure 2B is a typical spectrum for this type of nanoparticles (Garcia-Leis et al., 2013). Concentration of silver nanostars is typically 0.01 nM after synthesis (on the 50 mL reaction volume). After centrifugation and resuspension into 10% of the initial volume (5 mL), the concentration is 0.1 nM. The hydrodynamic diameter distribution of the synthesized silver nanoparticles was determined by NTA (Figure 2C), as average diameter of 217 nm (mode of 253 nm) and a standard deviation (SD) of 69 nm.

**Figure 3:**
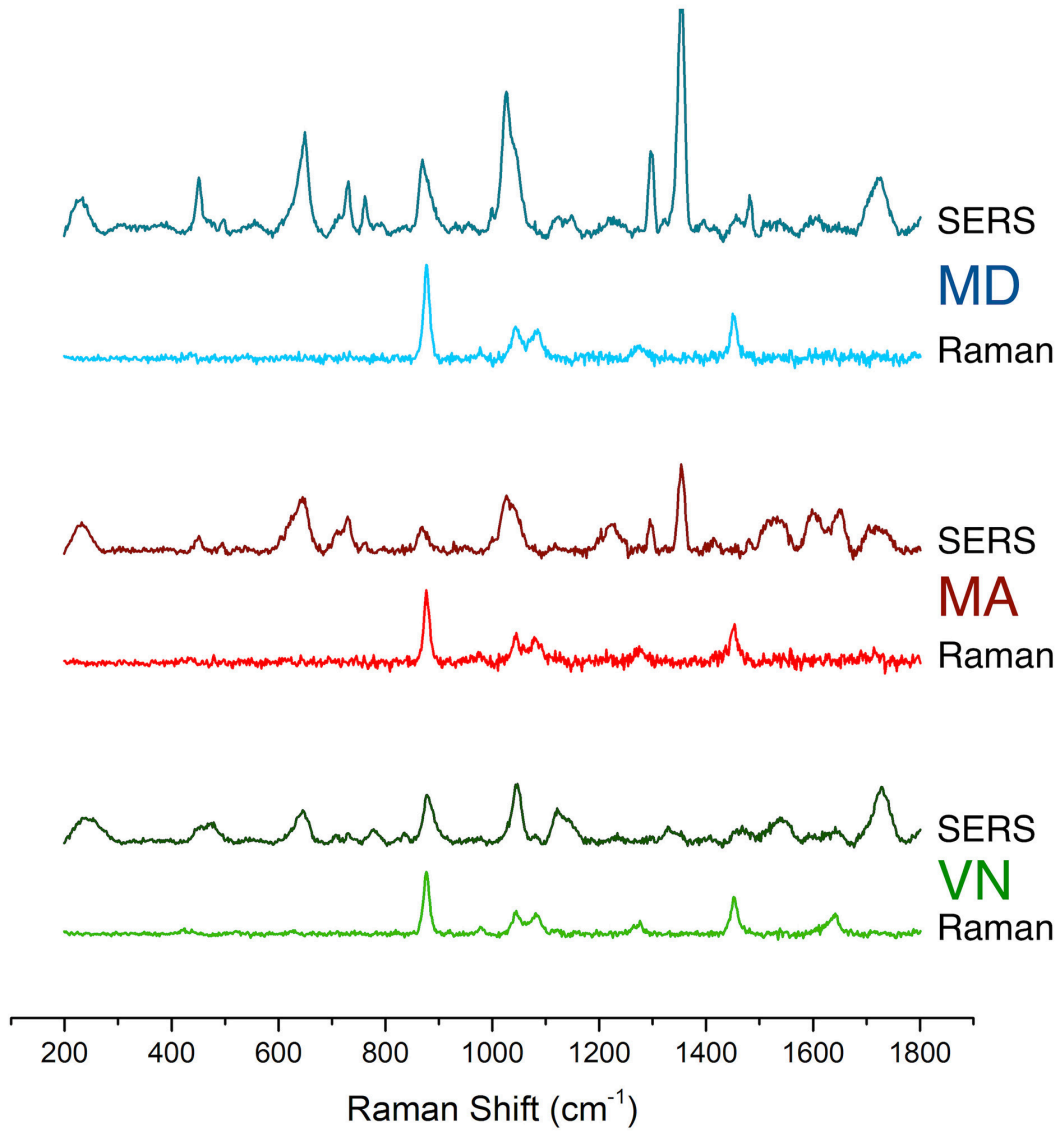
Normal Raman and SERS spectra for the three white wine samples of the *Maduro* (MD and MA) and *Verde* (VA) varieties, acquired using a 532 nm laser line. Spectra were baseline corrected using a polynomial subtraction procedure. Original spectra are in Supplementary Figure 3.

Figure 3 presents a comparison of normal Raman and SERS spectra for the three white wine samples, acquired using a 532 nm laser line. It should be pointed out that fluorescence background was removed from Raman and SERS spectra, using a polynomial subtraction procedure (Martin et al., 2015). Interestingly, the fluorescent background was much less intense in the *Verde* wine (VN), that in wines from the *Maduro* variety (MD and MA), suggesting that the *Verde* wine is poorer in fluorescent phenolic compounds when compared to *Maduro* wine. This is a plausible conclusion since the maturation of *Verde* wines is done in stainless steel containers, whereas for the *Maduro* variety, maturation is on oak wood barrels, sometimes with the addition of oak chips (Pérez-Coello et al., 2000; Afonso, 2002; Liberatore et al., 2010). These treatments, to improve aroma quality of the *Maduro* wines, lead to a high content in flavor active compounds, especially of the phenolic (fluorescent) kind.

Raman spectra of all three samples are very similar, with lines that can be attributed to the most abundant components, namely, sugars (namely fructose and glucose) exhibiting vibrations around 1,000–1,100 cm^−1^ (Ilaslan et al., 2015) and ethanol, showing at around 880 cm^−1^ (CC stretching) (Ilaslan et al., 2015; Martin et al., 2015). Another line appears at around 1,465 cm^−1^ in all the normal Raman spectra, that can also be attributed to ethanol, which is obviously found in abundance in wines, as mentioned above (Dolenko et al., 2015).

All SERS spectra obtained in the presence of AgNSs showed a strong enhancement of the Raman lines, with several new lines appearing (Figure 3). Also, lines from some groups of the identified molecules in Raman are missing or have very weak intensity, since SERS is a short-distance effect, only molecules close to the nanoparticles experience the strong electromagnetic field of “hotspots”. It is also interesting to notice that in contrast with normal Raman, SERS spectra from the three wine samples have notable differences between each other, hinting at an important role for SERS in wine fingerprinting. As SERS spectra are very complex and line attribution is nearly impossible, a statistical analysis by Principal Component Analysis (PCA) was thus applied.

Obtaining such intense and complicated SERS spectra for white wines mixed with citrate-capped AgNS, is probably related to several different types of molecules from the wine sample adsorbing to the surface of the AgNS. An interesting parallelism can be drawn to a study of the adsorption of different varieties of wines on membranes presenting different chemical functionalities (Mierczynska-Vasilev and Smith, 2016). In this study, the authors concluded that amine and carboxyl modified surfaces encourage the adsorption of constituents specifically from white wine as opposed to hydroxyl modified surfaces for rosé wine constituents, and acrylic acid surfaces for red wine. One could speculate that the carboxylic groups from the citrate capping are responsible for the extensive white wine components adsorption, resulting in the observed complexity of SERS spectra.

Another interesting feature observed for SERS spectra when comparing with normal Raman, is the significant fluorescence quenching induced by the AgNS. The presence of fluorescence emission in the normal Raman spectra and its quenching in SERS spectra, by the presence of AgNS, can be observed in the raw Raman and SERS spectra presented in Supplementary Figure 3. This effect was previously observed by some of us when the same type AgNS were adsorbed to an office paper substrate. In fact, the presence of AgNS on the surface of the paper was able to fully quench its intrinsic fluorescence emission, derived from excitation with a 633 nm laser line (Oliveira et al., 2017).

Two different protocols were used for SERS sample preparation, corresponding to different incubation periods, namely, overnight incubation and the “mix-and-read” approach, i.e., without incubation. Our rationale is based in the plausible adsorption process of the wine chemical components to the surface of the AgNS. In a process reminiscent of protein corona formation around metal nanoparticles, it is expected that wine chemical components with high abundance are firstly adsorbed on the surface, and over the time, they are replaced by components with lower concentration but higher affinity (Rahman et al., 2013). These lower concentration components, in the case of wine, are probably highly specific for each wine label, allowing fine fingerprinting.

**Figure 4:**
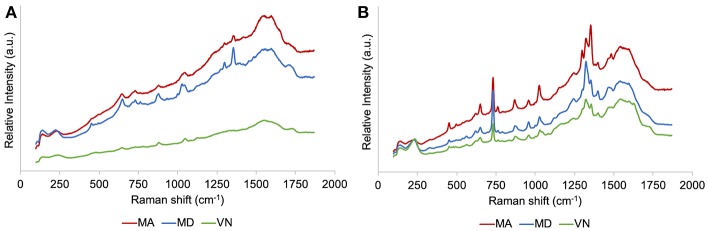
SERS spectra for the three white wine samples acquired using a 532 nm laser line. Two different protocols for sample preparation, corresponding to different incubation times, were used after mixing the AgNS colloid with the wine samples: **(A)** Overnight incubation, and **(B)** “mix-and-read” approach.

SERS spectra of the three wine samples under study, after simple mixing with the AgNS colloid, with no further treatments, are presented in Figure 4 for two different incubation periods. SERS spectra of samples that were incubated overnight (Figure 4A) show in general, much less resolution compared to SERS spectra of samples read immediately after the addition of silver nanostars (Figure 4B). On the other hand, comparing the three different wine samples for each incubation protocol, a better differentiation between samples is apparent for samples that were incubated overnight, compared to “mix-and-read” samples. In fact, the spectra of “mix-and-read” samples are individually very well-resolved, but look similar to each other, hinting at common and abundant wine components being the main contribution to the observed SERS signals.

In order to obtain the best fit for SERS data distribution and establish the principles of a fingerprinting method for the white wine samples, a statistical analysis of the SERS spectra was performed using PCA. This is the technique of choice for the intended data analysis, allowing compression of the data dimensionality at the same time reducing to a minimum the loss of information (Biancolillo and Marini, 2018). This analysis generates score plots which can highlight possible trends in the data, presence of clusters or of an underlying structure. Principal Components will have positive or negative scores on these plots and are directly correlated in the loadings plot with spectral regions with positive or negative loadings, respectively.

**Figure 5:**
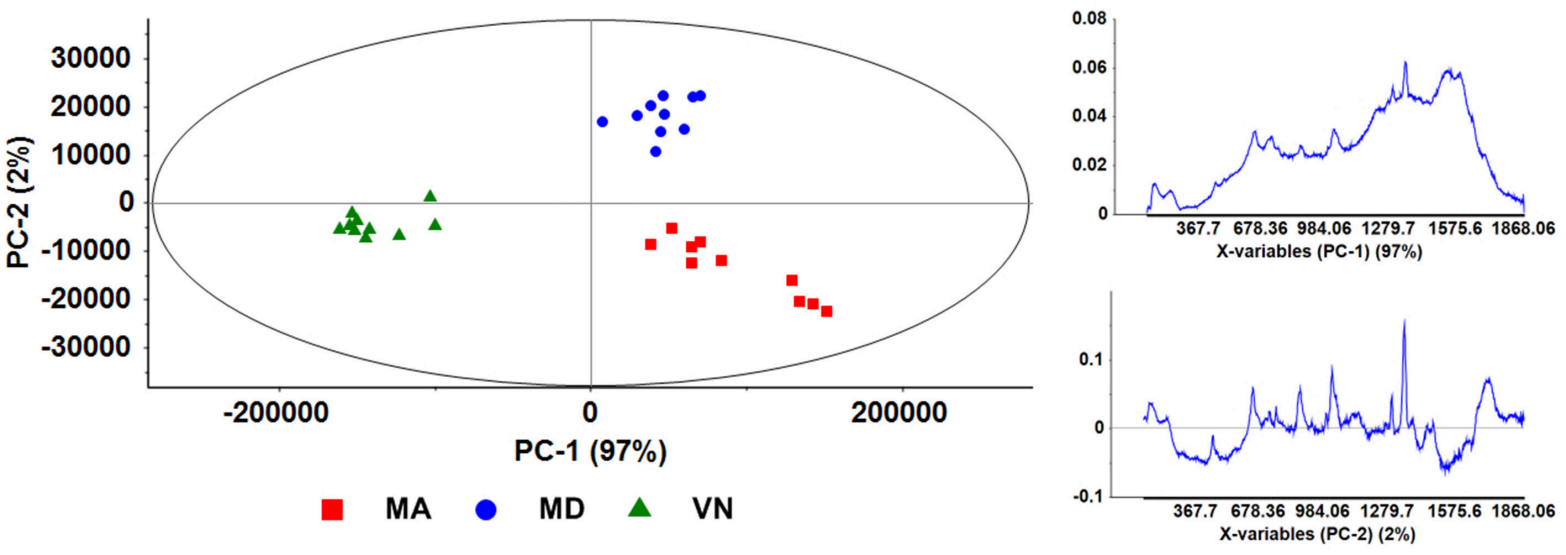
Scores plot depicting PC-1 and PC-2 (left); and loadings plots (right) for both Principal Components, PC-1 (top), and PC-2 (bottom). These plots are for SERS spectra obtained from samples prepared following the overnight incubation protocol, with data pre-treatment by BLC.

The score plot and loadings resulting from the PCA analysis with baseline linear correction (BLC) pre-treatment, of SERS spectra obtained by the overnight incubation protocol, are depicted in Figure 5. In the score plot, three data clusters are clearly distinguishable, corresponding to the three different wines. Furthermore, distinction between *Verde* and *Maduro* wines is possible from this plot, since it is related to principal component 1 (PC-1, horizontal axis), which has a 97% weight. In fact, *Verde* wine (VN) is clustered in the negative section of the score plot, while both *Maduro* wines (MD and MA) cluster on the positive section. The loading plot of PC-1 (Figure 5, bottom left), is an all-positive spectrum with a baseline that increases in intensity toward the high frequency region of the spectrum, an indication of sample fluorescence. This seems to be an important characteristic of *Maduro* wines, as these present a positive score for PC-1. This conclusion agrees with the hypothesis that the *Verde* wine is poorer in fluorescent phenolic compounds compared to wines from the *Maduro* variety.

The second principal component from the score plot of Figure 5 (PC-2, vertical axis, 2% weight) seems to essentially distinguish between the two regions of *Maduro* wine, with MD and MA clustering on the positive and negative sections of the PC-2 axis, respectively.

In data obtained for “mix-and-read” protocol, PCA analysis with data pre-treatment by BLC, shows a very important weight (91%) for PC-1. However, the samples score points are quite dispersed across the PC-1 axis, being both in positive and negative regions. It is nevertheless possible to recognize groups organized in the horizontal axis, so PC-2 seems to distinguish the three wines (Supplementary Figure 4).

**Figure 6:**
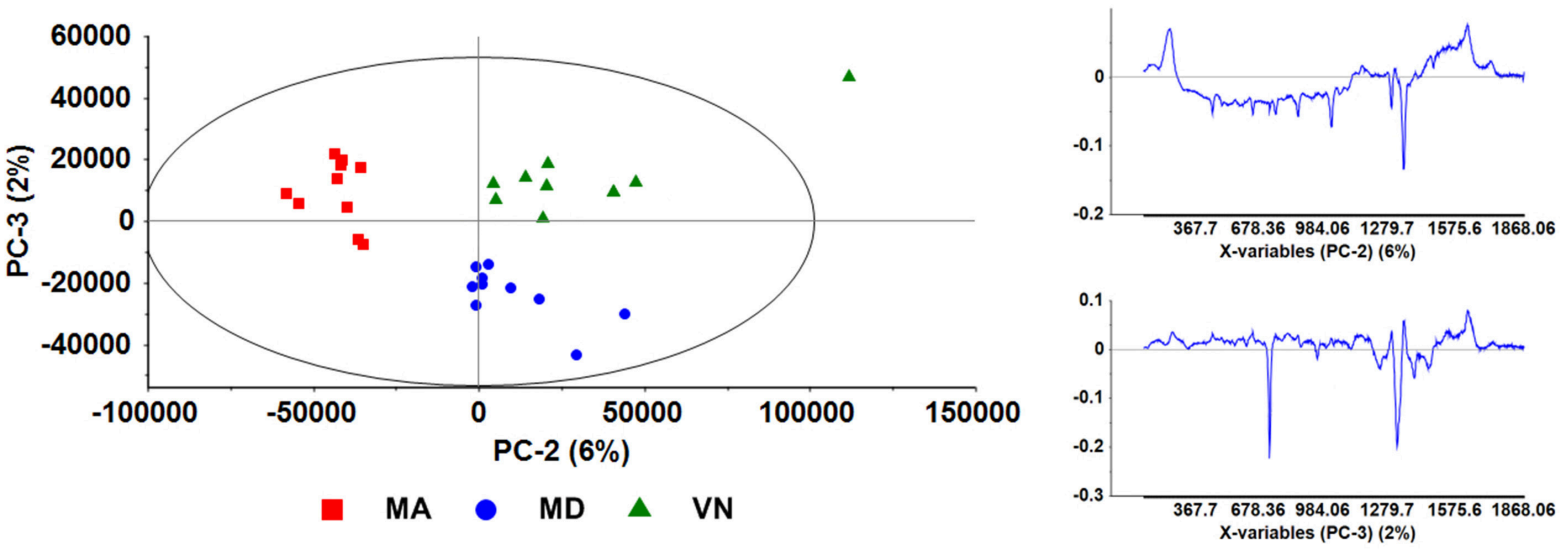
Scores plot depicting PC-2 and PC-3 (left); and loadings plots (right) for both Principal Components, PC-2 (top), and PC-3 (bottom). These plots are for SERS spectra obtained from samples prepared following the “mix-and-read” protocol, with data pre-treatment by BLC.

We then decided to produce a score plot of PC-2 (horizontal axis, 6%) vs. PC-3 (vertical axis, 2%) in order to evaluate its usefulness for the white wine samples discrimination (Figure 6). In spite of these principal components 2 and 3 presenting only a total weight of 8% of the total SERS data, their score plot clearly isolates three groups corresponding to the three white wines under analysis, thus allowing the intended discrimination between white wine samples.

This is a notorious case in which PC-1 loses relevance and PC-3 helps to differentiate wines. This is in line with what it was already discussed for this approach, specifically the determinant role of abundant and common wine compounds in the SERS signal. These common and abundant compounds are probably the main contributor for PC-1 in this case of the “mix-and-read” protocol, leading to a high distribution across the PC-1 axis and its high weight.

It is worth mentioning that applying the same PCA analysis to Raman spectra, gave no results as to discrimination of any of the three samples. PCA analysis was tried in the raw spectra and also using two types of data pre-treatment, namely BLC and SNV, in all cases with no discrimination results (data not shown).

In conclusion, the use of AgNS for wine fingerprinting by SERS was proven possible with these three wines. When a AgNS colloid is incubated with wine samples, SERS spectra obtained using two different incubation protocols, corresponding to two different incubation periods, allowed discrimination between white wines from two different Portuguese varieties (*Verde* and *Maduro*) as well as between wines of the Maduro variety from two different wine regions (*Alentejo* and *Douro*).

SERS signals derive from the adsorption of wine chemical compounds to the surface of the nanoparticles. These compounds exchange with AgNS capping agent, citrate and presumably become new capping agents. A capping agent is as close as possible to any nanoparticle surface, so the new capping comprised by wine-derived molecules will be greatly enhanced by AgNSs hotspots.

Either AgNS are functionalized quickly by the most abundant compounds in wine, that should be quite transversal to every wine, or they act as regular Raman enhancers, i.e., without direct conjugation, with sample components being under the influence of the hotspots electromagnetic field. This also will privilege the most abundant compounds, given the higher probability to be found in higher quantity near the hotspots.

It important to notice that a typical “mix-and-read” approach can be misleading, probably due to the emphasis given to abundant compounds in wine, that are not so specific from wine to wine. If long incubation periods of AgNS with the wine samples (e.g., 20 h) are used, wine compounds with higher affinity to silver surfaces will replace the abundant compounds. These low quantity compounds are probably more characteristic of each wine.

However, since the objective of this work was to find a fast, yet effective, method for wine fingerprinting, the “mix-and-read” protocol should be considered. If the weight of the most abundant species is somehow discarded (e.g., ignoring PC-1), it is possible to fully achieve the objective of obtaining results in a facile and expedite way.

Work is underway to add more wine samples in order to validate this method in a larger range. This will confirm the need of using a protocol focused in high affinity compounds or if a fast protocol is suitable for the purpose. Additional wine samples from more regions will also be analyzed, and besides different regions, different wine years can also be tested as a discrimination factor. With data from different years, it will be also possible to evaluate if there are some tendencies regarding a possible convergence or divergence of wine properties from different regions due to climate changes (Cunha and Richter, 2016).

## Data Availability

All datasets generated for this study are included in the manuscript and/or the Supplementary Files.

## Author Contributions

All the authors contributed equally to experiment planning. MA was responsible for experiment execution. MA and RF wrote the manuscript with contributions from EP and NL.

### Conflict of Interest Statement

The authors declare that the research was conducted in the absence of any commercial or financial relationships that could be construed as a potential conflict of interest.
