# Supplementary material for: Expedite SERS Fingerprinting of Portuguese White Wines Using Plasmonic Silver Nanostars
Source: Front Chem. 2019 May 24;7:368. doi: 10.3389/fchem.2019.00368 (PMC6543917; doi:10.3389/fchem.2019.00368)
Supplement: Supplementary file 1 [file Data_Sheet_1.pdf]

Supplementary Material

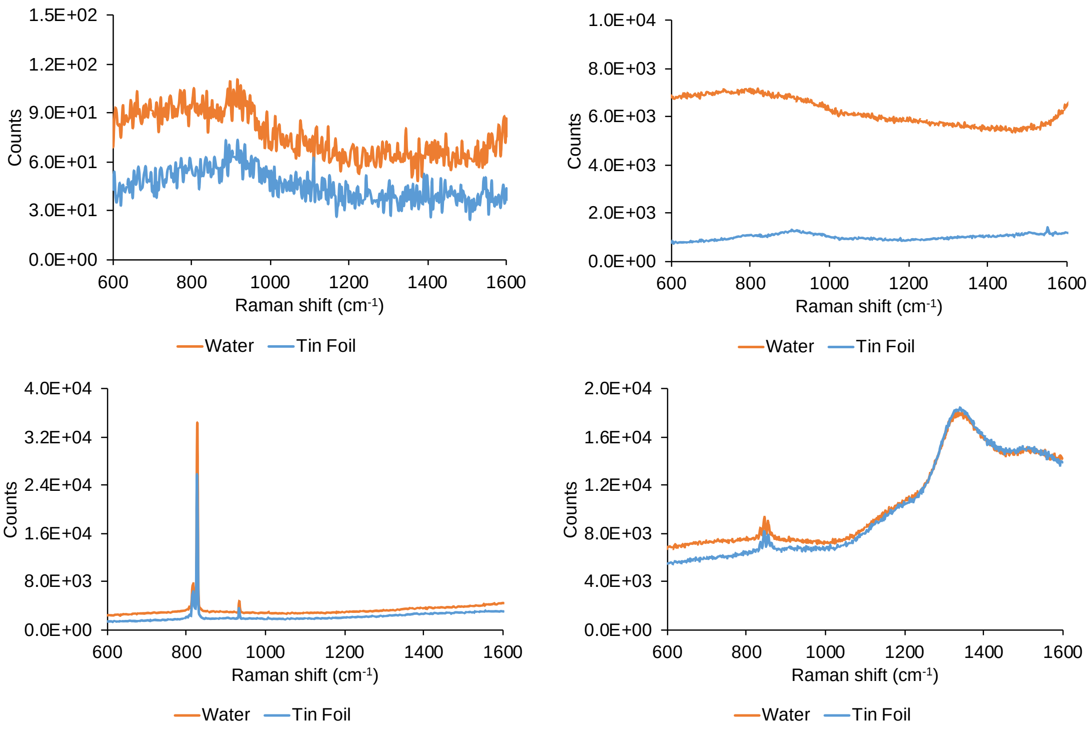

Supplementary Figure 1. Raman spectra for water (orange trace) and sample substrate, i.e., aluminum foil (blue trace), acquired using four different laser lines: 442 nm (top left), 532 nm (top right), 633 nm (bottom left) and 785 nm (bottom right) lasers. Sharp Raman lines are noticeable in the 825-830 cm^{-1} and 930-935 cm^{-1} regions, for spectra collected with the 633 nm laser. Also, for data collected using the 785 nm laser, there is a set of peaks in the 830-870 cm^{-1} and a broad band in the 1230-1430 cm^{-1} region, with two less intense bands in its vicinity. Regarding the support interference, the 442 nm and 532 nm laser lines seem to be the best options.

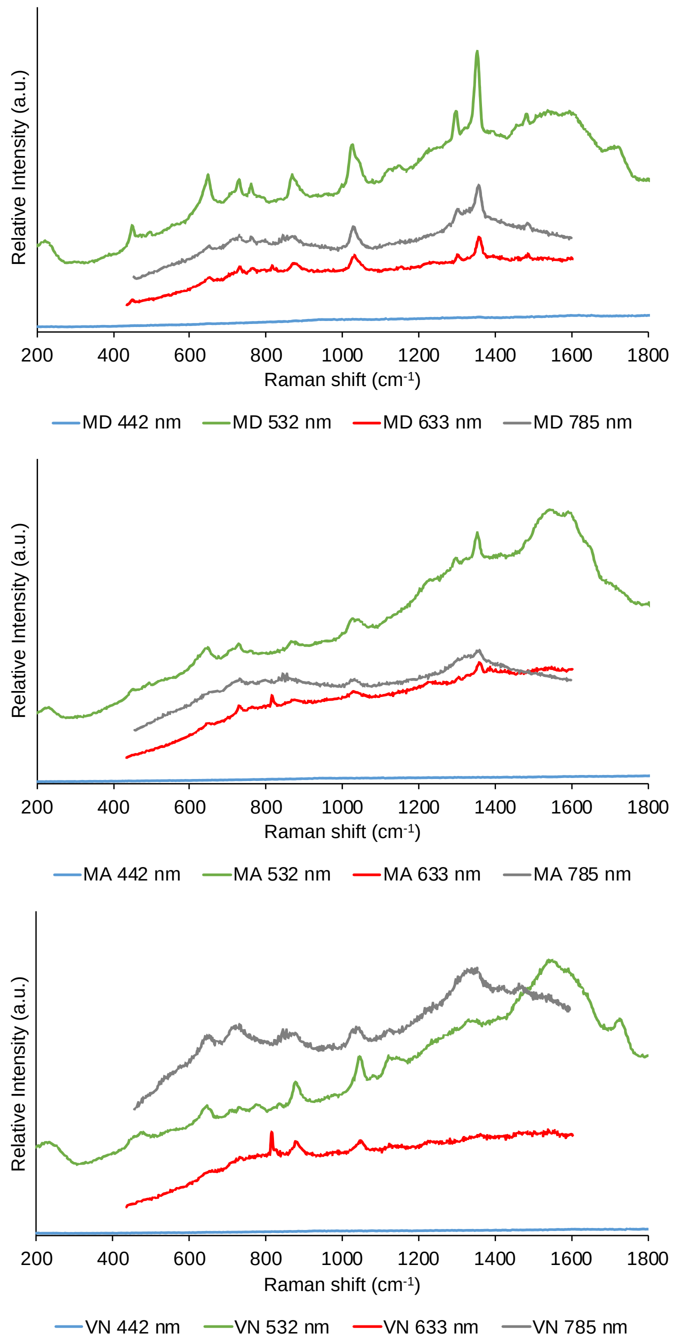

Supplementary Figure 2. SERS spectra for three white wines analyzed: *Maduro* from *Douro* (MD, top panel), *Maduro* from *Alentejo* (MA, middle panel) and *Verde* from the *Northwest* (VN, bottom panel), acquired using four different laser lines: 442 nm (blue trace); 532 nm (green trace); 633 nm (red trace); and 785 nm (grey trace). Interference signals from the substrate are noticeable around 850 cm^{-1} for the 633 nm and 785 nm lasers spectra, and also around 1350 cm^{-1} for the 785 nm laser (confirm in figure S1). Samples were prepared accordingly to the overnight incubation protocol (see Materials and Methods).

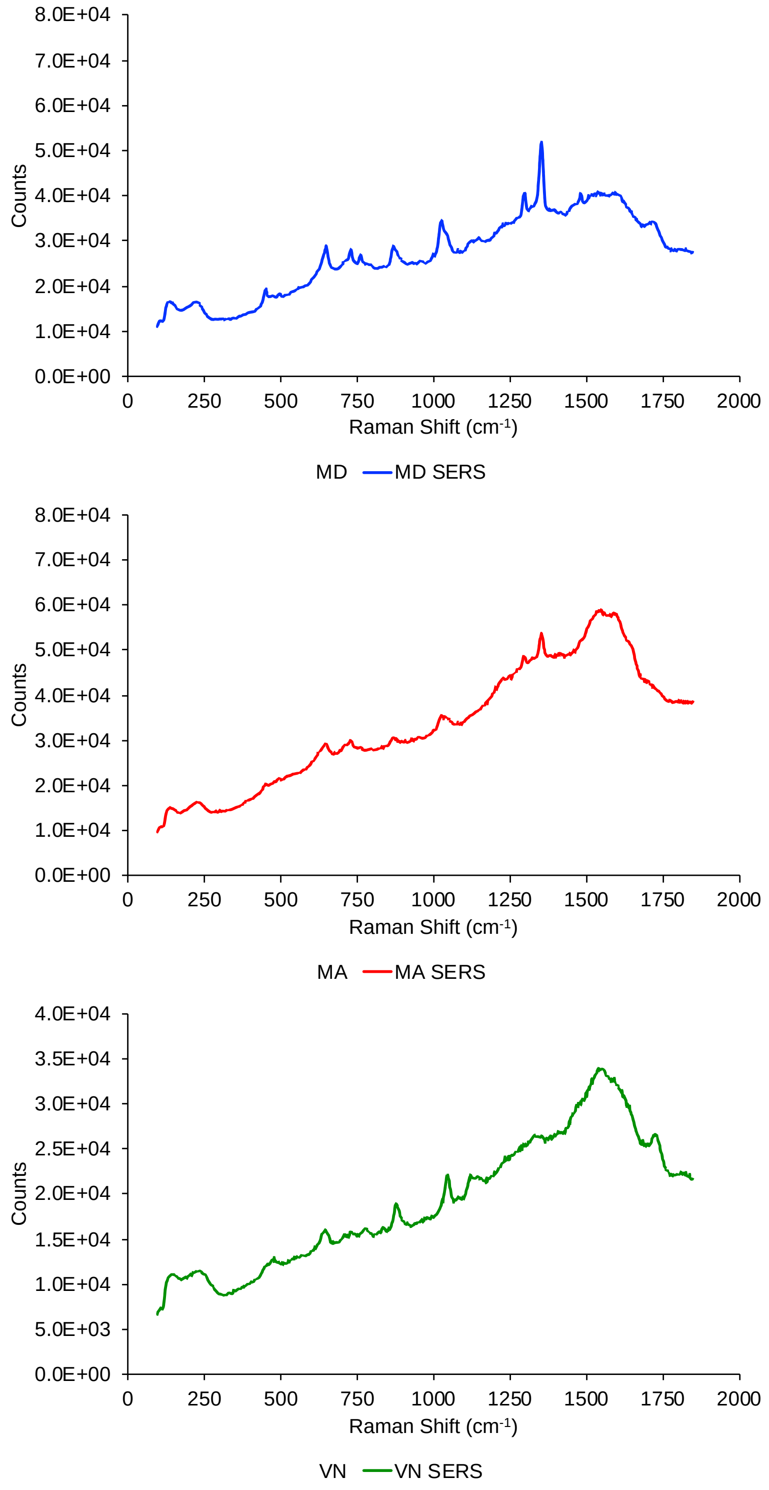

Supplementary Figure 3. Raw data used in in Figure 4 from the main text. Normal Raman (light colored traces) and SERS (dark colored traces) spectra for the three white wine samples of the *Maduro* (MD and MA) and *Verde* (VA) varieties, acquired using a 532 nm laser line.

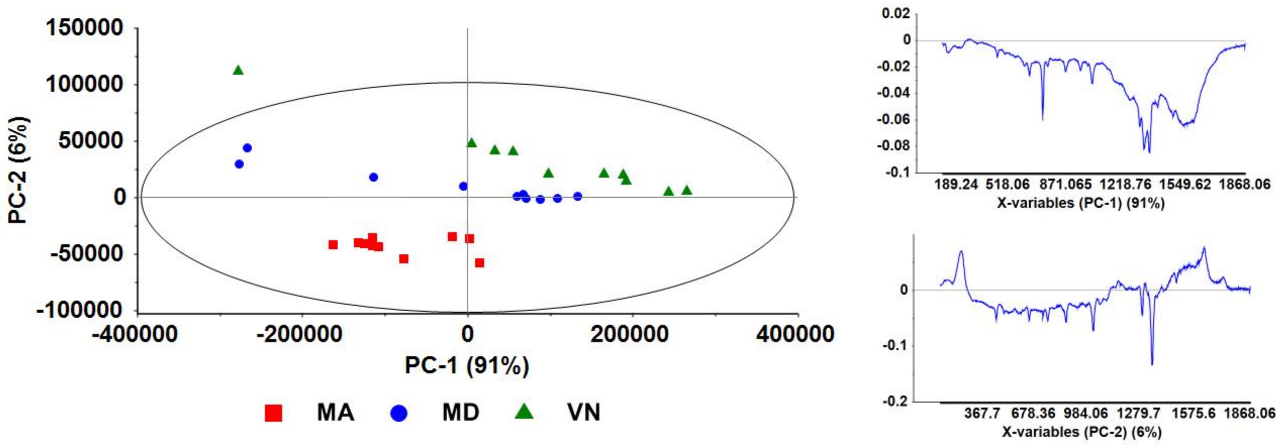

Supplementary Figure 4. Score plot depicting PC-1 and PC-2 (left) and loadings plot for the two PCs (right), applied to SERS spectra obtained from samples prepared following a "mix-and-read" protocol, with data pre-treatment by BLC.
